# Sustainability in quality improvement (SusQI): a case-study in undergraduate medical education

**DOI:** 10.1186/s12909-021-02817-2

**Published:** 2021-08-12

**Authors:** Philippa Clery, Stuart d’Arch Smith, Oliver Marsden, Kathleen Leedham-Green

**Affiliations:** 1 Bristol Medical School, University of Bristol, 1-5 Whiteladies road, Clifton, Bristol, BS8 1NU UK; 2 University Hospitals Bristol and Weston NHS Foundation Trust, Bristol, UK; 3 Centre for Sustainable Healthcare, Oxford, UK; 4 Medical Education Research Unit, Imperial College London, London, UK

**Keywords:** Medical education, Undergraduate, Sustainable healthcare, Environmental impacts, Quality improvement, SusQI

## Abstract

**Background:**

There is a pressing need for more sustainable healthcare. UK medical graduates are required to apply social, economic, and environmental principles of sustainability to their practice. The Centre for Sustainable Healthcare has developed a sustainability in quality improvement (SusQI) framework and educator’s toolkit to address these challenges. We aimed to develop and evaluate SusQI teaching using this toolkit at Bristol Medical School.

**Methods:**

We facilitated a SusQI workshop for all third-year Bristol Medical School students. We used mixed methods including questionnaires, exit interviews and follow-up focus groups to evaluate the outcomes and processes of learning.

**Results:**

Students reported: improvements in knowledge, confidence, and attitudes in both sustainable healthcare and quality improvement; increased self-rated likelihood to engage in SusQI projects; and willingness to change practices to reduce environmental impact in their healthcare roles. Factors for successful teaching included: interactivity; collaboration and participation; and real-life, relevant and tangible examples of projects delivered by credible role models.

**Conclusions:**

Students reported that SusQI education supported by the toolkit was effective at building knowledge and skills, and reframed their thinking on sustainability in quality improvement. Combining the two topics provided enhanced motivation for and engagement in both. Further research is needed on the clinical impacts of SusQI learning.

**Supplementary Information:**

The online version contains supplementary material available at 10.1186/s12909-021-02817-2.

## Introduction

The medical community has declared the impacts of climate change on human health as “the biggest public health threat of the century” [1]. NHS England has committed to net zero carbon emissions by 2040 [2] to mitigate the healthcare sector’s contributions to environmental degradation [3]. To achieve this, there is an urgent need for the healthcare workforce to be better equipped to engage in more sustainable healthcare practices.

The General Medical Council (GMC) mandates that newly qualified UK doctors are “able to apply the principles of sustainable healthcare to medical practice” [4, 5] and it is recognised that education for sustainable healthcare (ESH) has an essential role in attaining goals for sustainable development [6, 7]. However, there is currently very little ESH in medical curricula [8] and a lack of evaluative literature available to medical educators for embedding it [9].

**Fig. 1:**
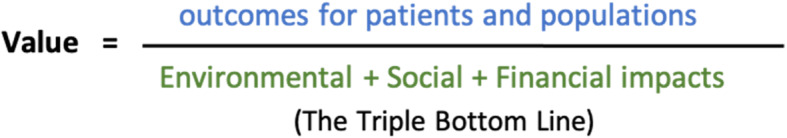
Sustainable value in healthcare. (Re-created from Mortimer, Isherwood and Wilkinson 2018, with permission)

Quality improvement (QI) offers an approach to ESH as it is inextricably linked with sustainability [10]. The NHS Sustainable Development Unit defines sustainable healthcare as “working across the health system and partners to deliver healthcare that delivers on the ‘triple bottom line’ i.e simultaneous financial, social, and environmental return on investment. It includes adapting how we deliver services, health promotion, more prevention, corporate social responsibility and developing more sustainable models of care” [11]. Improving the safety, efficiency and effectiveness of services has co-benefits on the triple bottom line (Fig. 1) by reducing unnecessary healthcare activity [12, 13]. QI techniques are therefore ideal for developing sustainable healthcare practices, as they provide established methods for designing, implementing, evaluating and embedding changes to services [14, 15].

Strategies for reducing the carbon footprint of healthcare focus on reducing the amount of healthcare that is needed, or on reducing the carbon intensity of services [16]. These include: health promotion; disease prevention; supported self-management; lean effective pathways; low carbon alternatives such as ‘greener’ inhaler and anaesthetic options; and attention to procurement and waste. Strategies for social sustainability in healthcare include improving: social equity; access to healthcare; safety and wellbeing for staff and suppliers; and attention to social determinants of health [17].

The recent AMEE (Association for Medical Education in Europe) consensus statement for ESH recommends a ‘sustainability in QI’ approach for developing a healthcare workforce that is not only informed about the interdependence of ecosystems and health, but also possesses the skills, values, capabilities to drive change and is motivated to foster change [7]. QI and sustainable healthcare have become priority areas for Health Education England [18], who have commissioned the Centre for Sustainable Healthcare (CSH) in collaboration with the Health Foundation to create a toolkit around the framework integrating sustainability with QI, a practice known as “SusQI”. SusQI supports practitioners to improve patient care whilst creating environmental, social and financial value (Fig. 1) [12, 13].

QI projects are a mandatory part of UK postgraduate medical training and the GMC requires graduating medical students to be competent in QI [4]. Medical students and trainee doctors are well-placed to identify inefficiencies and provide innovative QI solutions as they rotate frequently between departments and Trusts as unbiased observers of quality in care [19]. However, many trainees report feeling disengaged with QI [20]. Competing curricular priorities means that undergraduate QI teaching is often given little attention and students can perceive it as a tick-box exercise rather than a meaningful activity [21, 22]. SusQI therefore presents an opportunity to teach sustainable healthcare whilst enhancing motivation and engagement in QI.

The CSH’s free online toolkit has resources to support SusQI education [23] and is being piloted in UK medical schools. This is the first evaluative case-study of this pilot. We delivered SusQI teaching at Bristol Medical School and aimed to evaluate its impact on learning and motivation, and isolate key success factors for teaching delivery.

### Research questions

What is the self-reported educational value of SusQI teaching to participants?

What do participants describe as the key success factors of teaching delivery?

## Methods

### Methodology

This research is conducted within a pragmatic ‘real world’ paradigm, with educators as co-researchers, acknowledgement of real-world imperfections in data, and the creation of useful knowledge as our aim [24]. We have adopted Sandars’ recommendations for creating useful evaluative research: describing the intervention in sufficient detail to be replicated, and critically evaluating the processes in relation to outcomes [25]. We have used a mixed methods sequential explanatory design [26], including quantitative data from pre- and post-session evaluation questionnaires, and qualitative data from session feedback and follow-up focus groups.

### Research team

The research team comprised: two clinical academics involved in SusQI teaching and evaluation (PC, SdAS); a full-time educational researcher from a neutral institution (KLG); and a medical student who conducted focus groups (OM).

### Participants and context

Teaching took place in February 2020. Participants were third-year undergraduate medical students at the University of Bristol in the academic year 2019–20 (approx. *n* = 342). Neither QI nor sustainable healthcare had been taught formally as part of their medical curriculum thus far. There is a strong culture of sustainability in Bristol, particularly amongst the student population [27].

### Teaching intervention

**Fig. 2:**
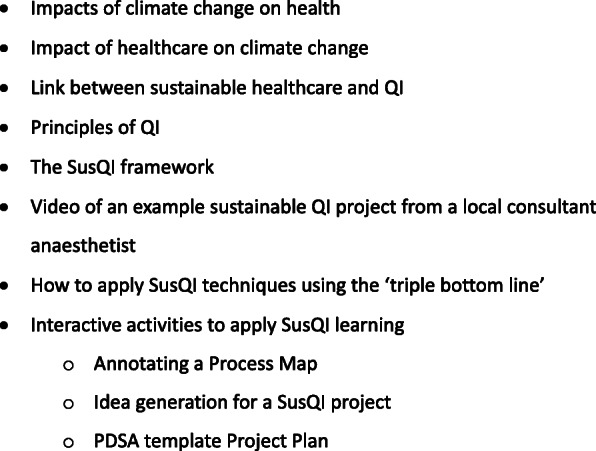
Topics covered in the workshop based on the SusQI toolkit

PC and SdAS designed and facilitated an interactive workshop based on the SusQI toolkit. Presentation topics are listed in Fig. 2 and workshop materials are in Additional File 1.

**Fig. 3:**
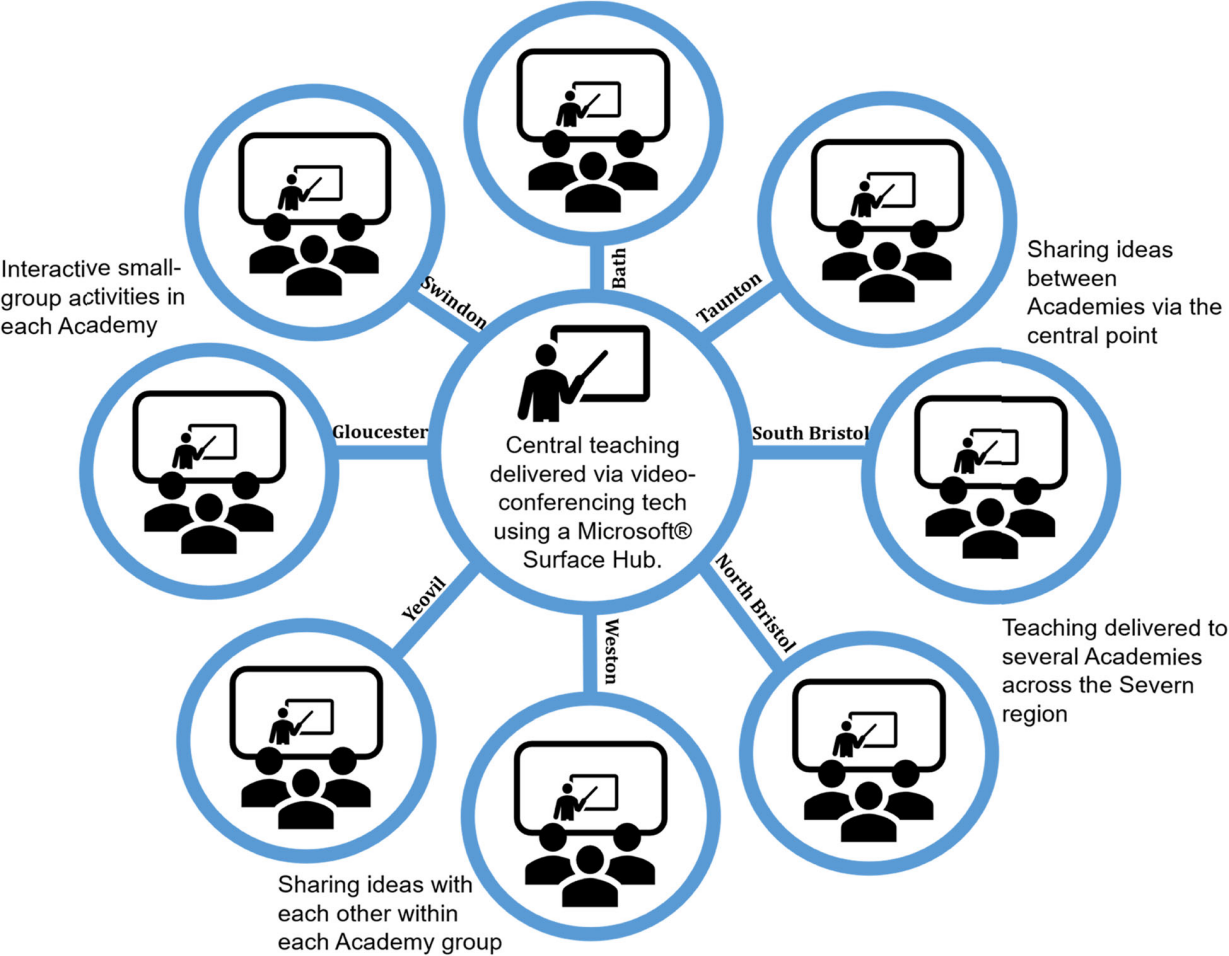
Hub and spoke model - depicts centralised teaching (hub) and teaching hospital Academies (spokes)

Teaching was delivered as part of the ‘Helical Themes’ curriculum, which addresses cross-disciplinary GMC learning outcomes and personal development. Themes are delivered as weekly ‘Hub’ sessions throughout year three and involve a centralised lecture followed by workshops, both delivered via video-conferencing software using Microsoft® Surface Hub technology. The workshops involve approximately 10–28 students in eight regional hospital trusts, or ‘Academies’ (Fig. 3) and include break-out activities via a communal interactive online whiteboard.

Prior experiences suggested that Hub sessions could be disengaging for students, so we paid particular attention to interactivity and included relatable case-studies to enhance engagement [28]. These included videos of local anaesthetic consultants describing their QI projects to reduce desflurane (a greenhouse gas) usage and recycle plastic facemasks.

Students were encouraged to plan or undertake a SusQI project following the session, but it was not mandatory. A clinical teaching fellow in each Academy was assigned to offer support; acting as a liaison with a network of doctors who had volunteered as SusQI project supervisors. We had planned a follow-up Hub session in May 2020 for students to share ideas and present projects, but this was disrupted due to the COVID-19 pandemic.

### Data generation

PC, SdAS and KLG developed an evaluation questionnaire (Additional File 2) informed by Kirkpatrick’s model of evaluation [29, 30], theories of motivation [31] and transformative learning [32]. The questionnaire invited participants to self-rate their baseline knowledge, confidence and attitudes, including how they valued learning about QI and sustainable healthcare. Baseline data was gathered a month before teaching, and attendees were invited to complete the post-session questionnaire immediately after teaching. The post-session questionnaire re-assessed the same domains, with additional questions to assess intention to undertake a QI project, and free-text questions to elicit session feedback. As part of an overarching evaluation of Hub teaching, students gave verbal feedback to an independent assessor when exiting the session. This was transcribed verbatim and provided to our research team as anonymised quotes. Five focus groups were conducted by OM three months after the teaching to evaluate the longer-term impacts of teaching. Participation was voluntary with informed consent. All potentially identifying content was redacted, and focus groups were facilitated by a researcher (OM) who was not in a position of power over participants. The University of Bristol provided ethical approval (#98065).

### Data analysis

Cronbach’s alpha was used to assess internal consistency and reliability of the questionnaire. Baseline and follow-up data were analysed to explore learning. Differences between students’ pre- and post-session scores were analysed using chi-squared tests. Qualitative data were coded into themes relating to outcomes, processes, and suggestions for improvement, facilitated by NVivo12 software. Outcome codes were subcategorised into domains of learning [33] and levels of outcome [29]. Codes relating to processes and suggestions for improvement were arranged into categories and themes using an inductive consensual process [34].

This paper presents an evaluation of the outcomes and processes of teaching. An analysis of the barriers and facilitators to application of learning in the clinical context is presented separately in a follow-up paper (Marsden O, Clery P, d’Arch Smith S, Leedham-Green K.: Sustainability in Quality Improvement (SusQI): challenges and strategies for translating undergraduate learning into clinical practice, unpublished).

## Results

The questionnaire had good internal consistency and reliability for each of the knowledge, confidence, and attitudes sections (Cronbach’s Alpha 0.86, 0.84 and 0.91 respectively).

One hundred and ninety-eight out of 342 students (59.7%) completed the pre-session questionnaire and 121 (35.4%) completed the post-session questionnaire. The session was compulsory but precise attendance was not recorded. One hundred and nine out of 121 (90.1%) students who completed the post-session questionnaire provided short-text feedback, and 15 out of 22 (68.2%) students from one Academy provided verbal feedback for Hub evaluation. OM conducted five online focus groups involving all 17 students that agreed to participate.

### Outcomes evaluation

**Fig. 4:**
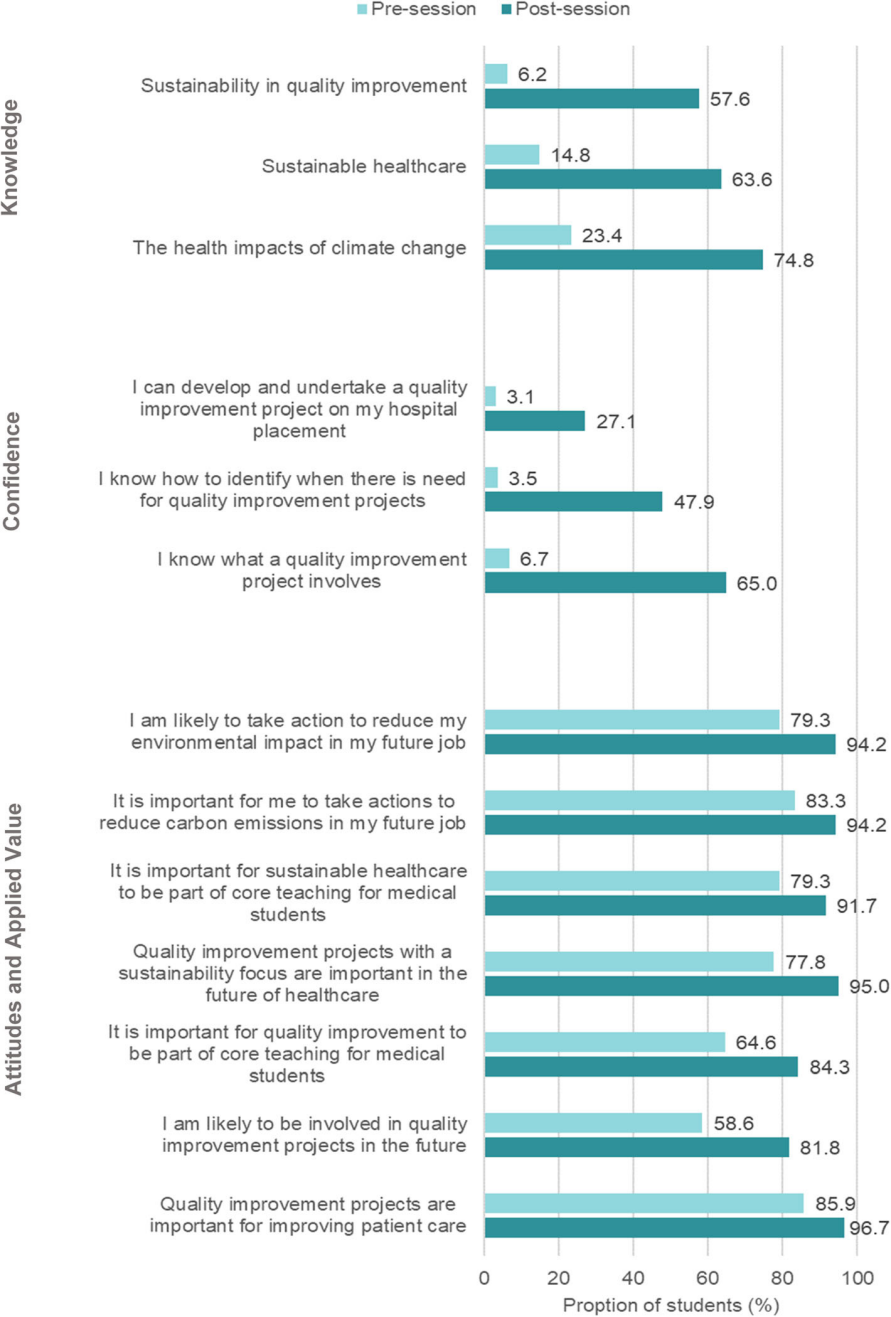
Student perceptions of SusQI learning needs and outcomes

The graph in Fig. 4 summarises pre- and post-session quantitative results. It presents the proportion of respondents answering positively or very positively on a five-point Likert scale.

Table 1 provides illustrative quotes mapped to Kirkpatrick’s levels of learning. Throughout, quotes are followed by a letter and respondent number: ‘A-E’ designates focus groups; ‘V’, verbal Hub feedback; and ‘Q’, questionnaire comments.

**Table 1:** Level of learning achieved by students based on Kirkpatrick’s (K) teaching evaluation pyramid

Kirkpatrick Level	Rating	Description	Implications	Illustrative quotes
K1: How did learners engage with the session?	**++**	Students valued the session, liked the resources and interactive group-based activities.	The teaching was well designed and delivered.	*“Definitely the best Hub [session] we’ve had so far.”* V9 *“It was something that I anecdotally told other people about because it kind of impacted on me so much.”* E3 *“It [was] great that there [were] so many resources like presentations and videos and worksheets”.* B3
K2: What did students learn? 1. Knowledge 2. Skills 3. Attitudes	**++**	Students developed knowledge and skills; were motivated; developed a ‘SusQI gaze’; their professional identity was congruent with SusQI values; they viewed the NHS as dynamic; saw quality improvement as valuable.	The experience built a new lens through which to view healthcare, and a triad of positive reframing.	Knowledge domain: *“I suppose you just want to think about the future and the fact that you want whatever you’re doing now it’s be available X number of years down the line, to make sure that [your resource] doesn’t run out. I think in the in the Hub session, we did talk about things like whether ambulances could be made more environmentally friendly, so carbon emissions, literally from vehicles, to hospitals. So it’s sort of thinking about all different aspects of the hospital rather than, like not just within the hospital.”* A3 *“You’re not just doing things for the sake of doing them or because people have always done it that way. You’re actually actively thinking about what is necessary and what isn’t and then what you can do to change that.”* D1 Skills domain: *“[You’ve got] that QI-head on your shoulders, [asking] what can I do? What can I improve? Why is this the way it is?”* C2 *“It did make me, kind of, more aware of the protocol for during one and you know that... That wheel... The um, “Plan study act”, whatever it was [PDSA cycle].”* C3 Attitudes domain: *“I always saw quality improvement as like, pretty dull and boring. And I just didn’t really care about it [but] quality improvement isn’t just about improving healthcare, it’s also about improving, like sustainability as well and bringing that into the forefront. I think that’s important because you’re gonna get a lot more people, a lot more doctors, also a lot more nurses and even porters, care more about sustainability, and I think it’s worth utilising that.”* D1 *“It’s just highlighted the fact that the NHS will be an ever evolving system, there’s always going to be something that doctors or students or anyone can do to improve it. And so I guess I’ll just be more on the lookout as I go through the ranks as to what things can be changed and what I can actually do to make it better.”* A3
K3: Did students apply (or intend to apply) their learning?	**+**	Few projects were undertaken. For some, motivated intentions could not overcome barriers.	The workplace environment does not naturally support students to enact SusQI	*“I spoke to my CTF (clinical teaching fellow) afterwards actually, and he sent me some... It was like a ‘e-learning’ thing about QI projects, which was really good. It’s sort of like, helped me get to grips with how to actually run one.”* D2 *“I remember feeling at the time quite inspired to... Well, I did actually get involved in a sustainability QI project... But it got squashed [by the COVID-19 pandemic] … We were sort of in the process of developing it … we were gonna tag on to the desflurane anaesthetic gas reduction project one in [name of hospital]. We were sort of gonna help spearhead that.”* D2 *“We were trying to do something in [name of hospital] but I think we’ve binned it because we got sent home obviously [due to COVID-19]. But we were gonna try and get more recycling bins around the hospital.” B4*
K4: Did applying their learning realise sustainable value for services?	**–**	No successful SusQI projects completed by the time of focus groups.	Significant improvements must be made before SusQI education creates impact on NHS sustainability.	N/A

### How did learners engage with the session? (Kirkpatrick level 1)

Of the 15 students from one Academy who provided verbal feedback upon exiting the session, all provided strongly positive immediate reactions. Participants described the session as *“a lot better than normal”* V8, *“better than average”* V10 or even *“definitely the best Hub [session] we’ve had so far”* V9. Respondents attributed this to the level of interactivity as well as the content, which was recounted as *“very relevant … current topics and things”* V13. Three students provided critiques in addition to their positive comments. Two felt it could have been even more interactive *“between the Hubs so like students talking to students, not just the central Hub”* V12 and one student said the session could have been *“a video tutorial that you can do in your own time” V15* instead.

### What learning happened? (Kirkpatrick level 2)

#### Knowledge domain

The proportion of respondents that reported ‘good’ or ‘excellent’ knowledge of SusQI, sustainable healthcare, and the health impacts of climate change all increased after the session (6.2 to 57.6%, *p* < 0.001; 14.8 to 63.6%, *p* < 0.001; 23.4 to 74.8%, *p* < 0.001, respectively). Participants demonstrated a deepening understanding of SusQI such as *“efficient and effective distribution of resources, so you can still provide same level care because you’re not wasteful”* D1. They reported that they understood the purpose of QI was to challenge and improve clinical pathways so *“you’re not just doing things for the sake of doing them or because people have always done it that way. You’re actually actively thinking about what is necessary and what isn’t and then what you can do to change that”* D1. Students discussed ways of reducing the carbon footprint of healthcare, including aspects of care that happen outside the hospital such as patient and staff journeys.

#### Skills domain

The proportion of participants reporting feeling ‘fairly’ or ‘completely’ confident in undertaking a SusQI project, knowing what QI involves, and identifying a need for QI increased by almost ten-fold in each domain (3.1 to 27.1%, *p* < 0.001; 6.7 to 65%, *p* < 0.001; 3.5 to 47.9%, *p* < 0.001, respectively). Students discussed how they had learnt to identify areas for improvement and use PDSA cycles for project planning.

#### Attitudes domain

Baseline data showed many students were already interested in sustainable healthcare prior to the session but were less interested in QI. Some described prior conceptions of QI as a mandated, tick-box activity: “*I thought QI was kind of something you had to do and I didn’t really see the huge importance of it*” D2. After the session, students reported a more positive attitude toward both sustainability *and* QI. 95% (from 77.8%, *p* = 0.103) ‘agreed’ or ‘strongly agreed’ that SusQI projects are important in the future of healthcare, and 94.2% (from 83.3%, *p* = 0.313) considered it important to take action to reduce carbon emissions in their future jobs as a doctor. 91.7% (from 79.3%, *p* = 0.045) and 84.3% (from 64.6%, *p* = 0.239) reported it was important for sustainable healthcare and QI teaching to be part of the core curriculum, respectively. However, differences were not statistically significant, meaning evidence for this change in attitudes is weaker than for other domains, and the finding may be unreliable.

Participants reported that the session built their awareness of how sustainability and QI are *“interlinked, rather than just separate categories of patient care”* C1, and it *“changed [their] impression of QI”* C2 as a valuable skill. Participants reflected on how SusQI provided enhanced motivation for both topics. Some described feeling more positive about their future role: *“I think a lot of people struggle with like “stagnation” in their jobs … It’s nice to still feel like you’re actually contributing and learning new stuff … I think that gives me a sense of meaning”* D1. Others felt more positive about the NHS which was seen as “*supportive of these changes*” whereas previously they had “*always thought of [the NHS] as set in their ways*” A1. Participants saw their generation as needing to lead change because *“[current] consultants didn’t have the education when they were at med school because it just wasn’t climate related”* E2.

### How did students apply (or intend to apply) their learning? (Kirkpatrick level 3)

62% of respondents reported they were ‘likely’ or ‘very likely’ to take part in a SusQI project following the session. There was also an increase in the proportion that said they intended to undertake a QI project in the future (81.8% from 58.6%, *p* = 0.014). Many described intentions to use what they had learned. In the post-session questionnaire, 40 out of 109 students proactively requested help with *“the organisational aspects”* Q122 of running a QI project, including *“how to approach someone with regards to starting a QI project”* Q105 and *“who to contact, forms to fill out”* Q19. Some went further, asking for help in *“implementing change following a QI project”* Q51 and *“support in publishing a project”* Q16*.* In the follow-up focus groups, two out of 17 students described attempting SusQI projects which related to the case-studies that had been presented (recycling and anaesthetic gas reduction). Projects were interrupted however by the COVID-19 pandemic. Others described personal and institutional barriers to getting started on projects, which are discussed in our follow-up article (Marsden O, Clery P, d’Arch Smith S, Leedham-Green K.: Sustainability in Quality Improvement (SusQI): challenges and strategies for translating undergraduate learning into clinical practice, unpublished).

### Process evaluation

Students reflected on what was valuable about the processes of the teaching session, how it facilitated learning, and gave suggestions for improvement. Our themes are summarised in Table 2.

**Table 2:** Thematic structure presenting key factors for successful QI teaching and suggestions for improvement

**Factors that enabled learning**	1. Interactivity and Participation	*1.1 Role of Hub technology*
		*1.2 Engaging in critical discussions with peers*
	2. Content	*2.1 Pitched at the right level*
		*2.2 Balance between shock and hope*
	3. Real-life	*3.1 Relevance to practice*
		*3.2 Examples of achievable projects by near peers*
**Suggestions for improvement**	4. Additional support outside of the teaching session	*4.1 Having resources for action*
		*4.2 Balance ‘ready-to-go’ project ideas with student choice*
	5. Adapting the teaching structure to suit student needs	5.1 *Curricular positioning and emphasis*
		*5.2 More interactivity*
		*5.3 More than one session*
		*5.4 Sustainability integrated consistently across the curriculum*

### Role of hub technology

Students discussed two types of interactivity that were important for this session: feeling engaged with video-linked teaching itself; and interactivity and participation with others. They thought this session *“was one of the best [Hub sessions] because there was so much opportunity for [them] to get involved”* V5*.*

Hub video-conferencing technology was described by some as a barrier to learning as it *“doesn’t engage students”* E1. Teaching via a screen *“removed”* a sense of importance from the topic or induced a sense of dissociation *“like it never happened”* E2 despite willingness to engage. Students identified interactive participation as a key factor in overcoming this. They felt *“[sessions] are better when they’re interactive and get participation on [the student] side of the Hub”* V5 and it was important to *“do stuff in the Academies rather than just watching a screen”* V8.

### Engaging in critical discussions with peers

Interactivity not only enhanced enjoyment and learning, but also facilitated networking with like-minded peers. Break-out groups were described as a platform to build links with “*other medical students [who] actually do care about this as well*” C3. Discussions facilitated a collective confidence to question the status quo of unsustainable healthcare and to socially construct SusQI project ideas that they would otherwise not have considered.

*It’s hearing everybody else’s ideas and then you get from other people “Oh yeah I hadn’t thought about that, but that’s, that’s a good point, that makes sense!”.* C1

Participants described how the session broadened their understanding of ways in which they could improve sustainability, as well as their confidence in leading change. They reported discussing a *“combination of lots of different avenues”* A2 and found it *“quite empowering”* C2.

*It’s not just the “higher-ups” that can make changes … you yourself, you can make small changes that can make a difference.* C2

### Pitched at the right level

Several said the session was *“most useful”* because it was *“on [their] level”* V11 of understanding. SusQI was a novel topic that was not taught elsewhere in the curriculum, and therefore it was important to *“gauge [their baseline] knowledge”* V14 and deliver teaching that was not too specialist nor reliant on extensive pre-reading, because a lot of students “*hadn’t done [the pre-reading] for any Hub session*” D1. The session was seen as an introduction to SusQI which supported them in *“having an awareness of the topic, rather than being experts in it”* D1.

### Balance between shock and hope

Participants found statistics on climate degradation *“scary”* D2 but were grateful that they were balanced with hope for change. They commented on how impactful the session was in communicating the need for action. Some concepts like carbon emissions were described as intangible, *“you can’t physically see the changes going on around you”* A2, so students valued information that was presented in a way that they *“could visualise quite easily”* D2.

*I think on the slides … it was something like, if [the healthcare sector] was a country, it’d be the fifth biggest CO*_*2*_*emitter in the world... [examples] like that really sort of put it in perspective.* A2

Participants appreciated concrete examples of solutions and said that SusQI provided a *“focus on action”* D2 and built hope for the future, preventing students from feeling *“there’s nothing [they] can do to help”* or *“becom [ing] disillusioned with everything”* D2. The consultant project examples helped frame the NHS as a flexible institution with *“people [who are] willing to change”* A1, and the session provided the optimism they needed to engage.

*It wasn’t too depressing, like there was like an element of hope … we have the capability to make a massive difference, and in that respect, [the session] was quite inspiring.* A1

### Relevance to practice

Participants described the need for teaching to be relevant and congruous with current practice. They said teaching should *“fit in with what [they’re] doing [on placement]”* V7 and *“actually affect [their] practice”* C4, otherwise they would disengage.

*[it needs to be] put in practice when we’re actually out there on wards because during the Hub session it just feels a bit removed from actual clinical learning, but it is all applicable.* E2

Linking teaching to GMC outcomes helped to validate the session in the eyes of learners: *“the GMC outcome things at the end are quite good as well, just because it seems like people at the top [of medical leadership] say you need to know this. So, you should know this kind of thing”* E1.

### Examples of achievable projects by near peers

Participants valued real-life examples of SusQI projects. They wanted *“realistic ideas”* Q110 and to *“hear how other people’s projects have been”* Q24. Many said they *“really enjoyed the videos”* of local clinicians presenting their SusQI projects and thought they *“were a great way to start the Hub session”* A3 because they quickly captured attention, were engaging and had long-lasting impacts:

*… the video around the gases, I definitely engaged with and that stuck with me. It was something that I told other people about because it kind of impacted on me so much.* E1

They described feeling reassured seeing senior SusQI *“role models”* D2, which encouraged them to approach consultants about projects.

*I think … I could do that and I could talk to somebody more senior about it.* C1

### Having resources for action

Students reflected how the activities from the toolkit, which included annotating a process map and creating a PDSA cycle plan (see Additional File 2), gave them the skills and resources to apply their learning *“through a systematic approach”* V7. Online resources that they could refer back to were valued: *“it [was] great that there [were] so many resources like presentations and videos and worksheets”* B3.

The Hub session provided preliminary ideas and inspiration, but some felt the *“nitty gritty of ‘this is how you do a PDSA cycle’ [is] better self-taught”* D2 and requested resources for self-directed learning. Some felt that the session only scratched the surface of SusQI and that students *“need more knowledge”* C2 before conducting projects themselves.

### Balance ‘ready-to-go’ project ideas with student choice

Students appreciated ‘ready-to-go’ project ideas but felt these needed to be balanced with student choice:

*If [the Hub teachers] had given us maybe a broader range of [projects] … it might have sparked more people’s interests because I suppose everyone’s interested in different things.* A3

### Curricular positioning and emphasis

Students felt the single session did not reflect the importance of the topic: *“one talk about sustainability and quality … and that’s it? And then you move on to the [Hub session the following week] about something completely different”* C4. Friday afternoons were described as a grave-yard teaching slot, with some students distracted or not attending teaching at all. They thought *“it was a shame”* because even if the topic was interesting, the setting limited engagement and then they would *“go home at the weekend and forget about it”* E3.

### More interactivity

Students suggested even greater interactivity. This included bringing SusQI into their case-based-learning *“integrating it as part of one of the cases rather than as a Hub session so that you can actually get that discussion”* B1, or to incorporate a *“Q&A session”* with SusQI project experts *“so [they’re] able to ask that person about limitations in the project and kind of how [a medical student] could really go about it”* C2.

### More than one session

Students expressed a desire for more than one session. One suggested *a “spaced repetition kind of model of learning”* D1. Another suggested integrating sustainability into the curriculum as a spiral cross-cutting theme to ensure important messages were consistently conveyed.

*Had there been more sessions, making this a more kind of long-term [teaching] … it just gets in your head a little bit more, rather than just a one off session, you know.* C1

Another suggested splitting the session into a *“two part thing”* B1, either for teaching QI separately before integrating it with sustainability, or having a follow-up session with clear project explanations and opportunities to share ideas and ask questions: *“in a next session that could be someone who’d done their own project and they can answer your questions”* C2. Participants wanted time between sessions to do their own reading and discuss project ideas.

*If we were kind of having that week gap and knowing that we would have the second session … , it would motivate people a bit more to look at [the pre-reading] and actually do some work around it.* C1

Some felt that learning two new topics at once (sustainable healthcare and QI) was challenging and suggested concepts of sustainable clinical practice needed to be integrated more broadly across the curriculum, and not met for the first time in QI teaching.

*I.. my brain sort of compartmentalised sustainability being one thing and QI being another thing, but it was a great introduction to QI.* B3

### Sustainability integrated consistently across the curriculum

Confirming the need for spiral, cross-cutting sustainability teaching, one participant reported a lack of information about the environmental impacts during their anaesthetics teaching, which created conflicting messages:

*He was going on about how great these gases were and I was like “yeah but, you haven’t touched on the impacts on the environment”* E4

Others asked for better integration of sustainability into other parts of their teaching, and for the curriculum to bridge the gap between workplace practices and idealised practices so *“it could be solidified in [students’] brains and put in practice when [they’re] actually out there on the wards”* E2.

## Discussion

We have presented an evaluation of SusQI teaching for third-year Bristol Medical School students, analysing the impact on their learning, exploring what made the session valuable, and how it could be improved. Our findings support our theory that linking sustainability with QI in the form of SusQI education, facilitated through an online Hub model with interactive local workshops, was effective at building motivation and skills, and reframing thinking on sustainability in QI, within this participant sample and context. Participants reported improvements to their knowledge, confidence, and attitudes in both topics, corresponding to a self-rated likelihood to engage in SusQI projects and willingness and intention to change practices to reduce environmental impact in their future healthcare roles.

Interactivity and participation were reported as key factors in the teaching’s successful outcomes, particularly peer discussions and collaborative activities. Video-conferencing and Microsoft® Surface Hub ‘Whiteboard’ technology facilitated this but needed to be utilised thoughtfully and appropriately, with sufficient opportunities for interactivity and active engagement, to prevent students feeling disengaged or disassociated. The COVID-19 pandemic has highlighted the need for equity, adaptability and community in video-linked teaching [35].

Climate change has been associated with eco-anxiety in student-aged populations [36]. Our workshop was successful in balancing shock with hope by proposing achievable solutions to problems. Participants described feeling inspired and empowered rather than anxious or downhearted which, given current concerns about student wellbeing [37], is important.

Another finding was the importance of real-life applicability and relevance of teaching to students’ curriculum and careers. Our research confirms previous findings that suggest students gain inspiration from examples presented by relatable clinicians [22] and where teaching is facilitated by clinicians who are passionate about QI [31].

Previous research demonstrates that trainees engage more in QI learning when they design a project to address real-life situations that are important to them and actively participate in finding solutions to those problems [38]. We found that participants reported that linking sustainability to QI directly supported their engagement in QI teaching.

Participants saw curricular positioning as an indicator of the importance of the topic of sustainability. There is a disconnect between what the chief executive of NHS England describes as an “emergency”, that, if unabated, “will disrupt care, and affect patients and the public at every stage of our lives” [2], and current space for it in curricula. Whilst competing educational interests make it difficult to add topics to already overcrowded curricula, students felt that sustainable healthcare concepts should be integrated within existing teaching across the curriculum, and that SusQI teaching needed follow-up sessions for troubleshooting and showcasing project work.

New subjects can be challenging to introduce due to a lack of expertise amongst teachers and difficulties releasing clinicians for faculty development [39]. The video-linked Hub software proved invaluable as it allowed experts to teach both students and teaching fellows across eight teaching hospitals simultaneously. Previous research suggests that new topics can be effectively disseminated to clinical teachers through partnership approaches [40], including ‘trickle up’ learning as students discuss projects with their supervisors [41]. This model could therefore be effective for engaging both undergraduates and their clinical teachers, thus accelerating the development of a critical mass of SusQI-educated health professionals.

Our findings suggest the key recommendations for implementing medical student SusQI teaching include: high levels of interactivity facilitated by individual reflection and group activities; allowing for discussion amongst peers to develop confidence and generate ideas; gaining an appreciation for students’ level of understanding and engagement of sustainable healthcare prior to the session; balancing ‘fear’ of impacts of climate change on health with resources for action; and including real-life examples of QI projects from local colleagues.

### Strengths and limitations

This is the first evaluation of the SusQI toolkit in undergraduate medical education. Teaching was delivered as part of core teaching which enabled us to survey many students and to gain insights from students that might not have electively chosen to learn about SusQI. The combination of qualitative and quantitative enabled a ‘clear box’ evaluation; not only do we report whether the session worked or not, we can illuminate *why.* The main limitation is self-reported outcomes data and inability to directly assess impacts (i.e., undertaking a SusQI project) due to the COVID-19 pandemic hindering students’ opportunities in clinical practice between February and May 2020. Other limitations include optional feedback so those who were less engaged may not have responded, and not all 342 students will have attended teaching despite it being compulsory. Therefore, we cannot be certain about the questionnaire response rate. If we assume all students attended the session, only 35.4% completed post-session questionnaires, introducing response bias in favour of those who enjoyed and engaged in the session. Lastly, participants were from one UK university in a city that champions environmental sustainability [27], therefore extrapolation more widely may not be possible.

## Conclusions

Introducing environmental and social pillars of sustainability into QI teaching was reported to assist engagement and learning in QI education in this sample of third-year Bristol medical students. The hub and spoke model offered a novel and interactive way to deliver standardised teaching to geographically distributed teaching hospitals. It also allowed experts to telecast to multiple workshop sites, reducing the need for local faculty development. Key factors for successful SusQI learning included: interactivity; opportunities for collaboration and participation; and real-life, relevant and tangible examples of successful projects delivered by credible role models. Students felt the SusQI toolkit was pitched at the right level but asked for extra resources for actual project work and wanted more information and support on how to get started. They called for sustainability to be integrated across the curriculum and not siloed within QI teaching. We recommend ongoing evaluation of SusQI initiatives nationally that follow-up student projects, to assess longer-term impacts in practice (particularly once COVID-19 restrictions are not a barrier for students engaging in SusQI projects), to identify factors for effective SusQI education in other contexts and to assist rapid dissemination.

This study suggests that the SusQI toolkit created strong intentional value: students learned skills that they wanted to apply. Further research is needed to explore barriers and facilitators to translation of learning to the clinical context, as well as the impacts of projects on the sustainability of healthcare services. Our follow-up paper (Marsden O, Clery P, d’Arch Smith S, Leedham-Green K.: Sustainability in Quality Improvement (SusQI): challenges and strategies for translating undergraduate learning into clinical practice, unpublished) addresses this first question.

## Supplementary Information

**Additional file 1.**

**Additional file 2.**

## Data Availability

The datasets used and/or analysed during the current study are available from the corresponding author on reasonable request.

## Abbreviations

SusQI: Sustainability in quality improvement
GMC: General Medical Council;
QI: Quality improvement
NHS: National Health Service.
