# Supplementary material for: Sustainability in quality improvement (SusQI): a case-study in undergraduate medical education
Source: BMC Med Educ. 2021 Aug 12;21:425. doi: 10.1186/s12909-021-02817-2 (PMC8358256; doi:10.1186/s12909-021-02817-2)
Supplement: Supplementary file 1 — Additional file 1. [file 12909_2021_2817_MOESM1_ESM.docx]

**Sustainability in quality improvement (SusQI): a case-study in undergraduate medical education**

Philippa Clery^1,2^, Stuart d’Arch Smith^3^, Oliver Marsden^1^, Kathleen Leedham-Green^4^

^1^ Bristol Medical School, University of Bristol, Bristol, UK

^2^ University Hospitals Bristol and Weston NHS Foundation Trust, Bristol, UK

^3^ Centre for Sustainable Healthcare, Oxford, UK

^4^ Medical Education Research Unit, Imperial College London, London, UK

*Workshop slides* (visit susQI.org for updated generic template session)


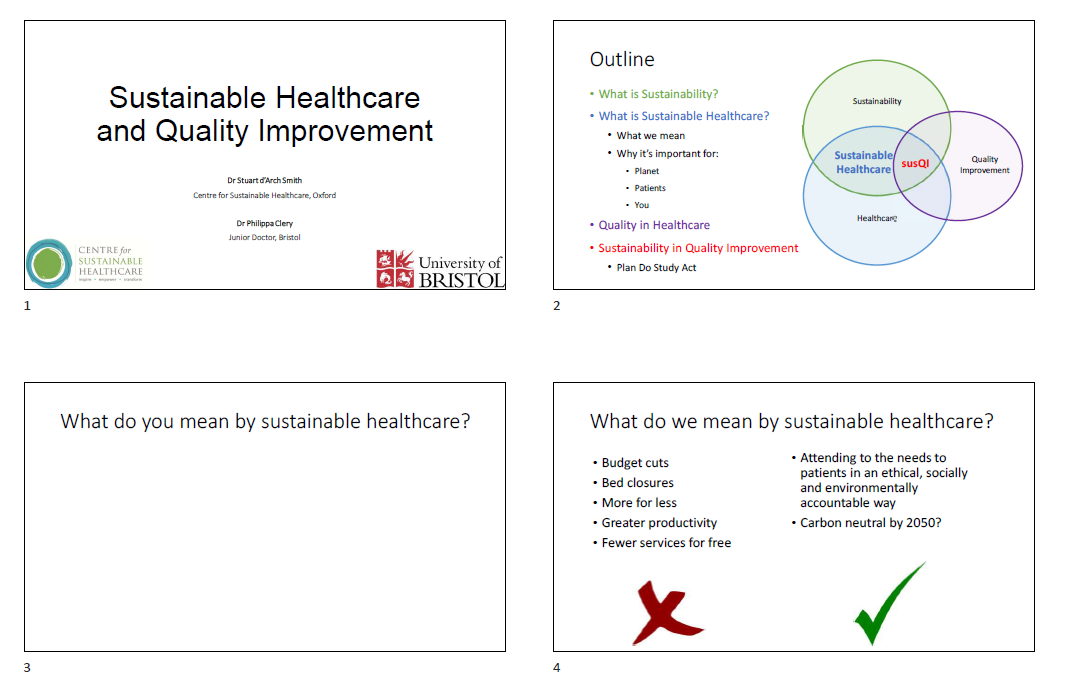


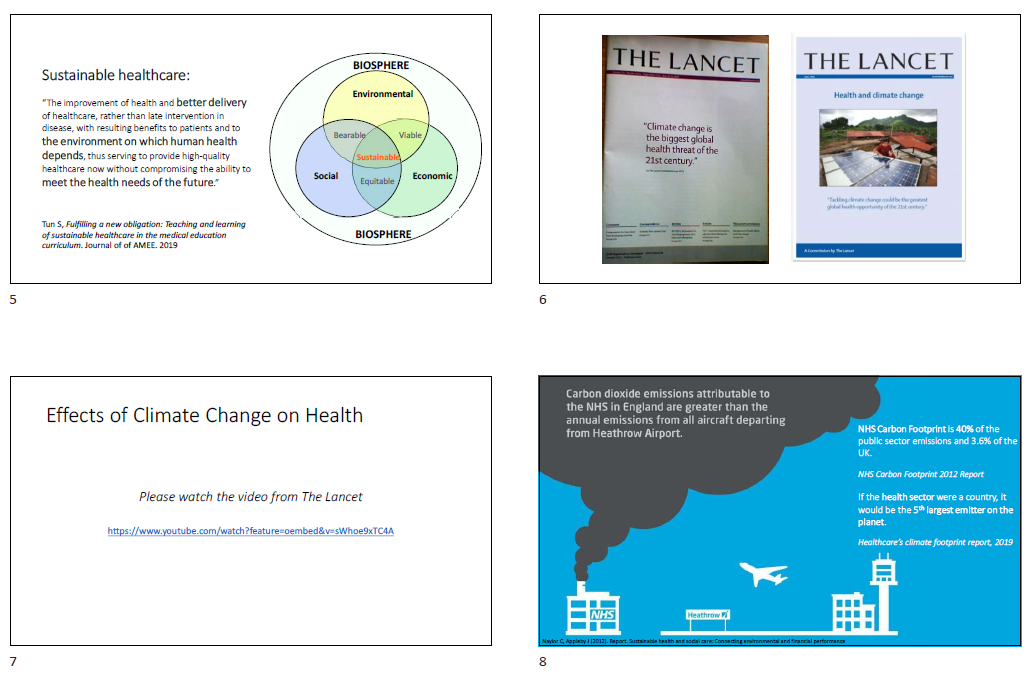


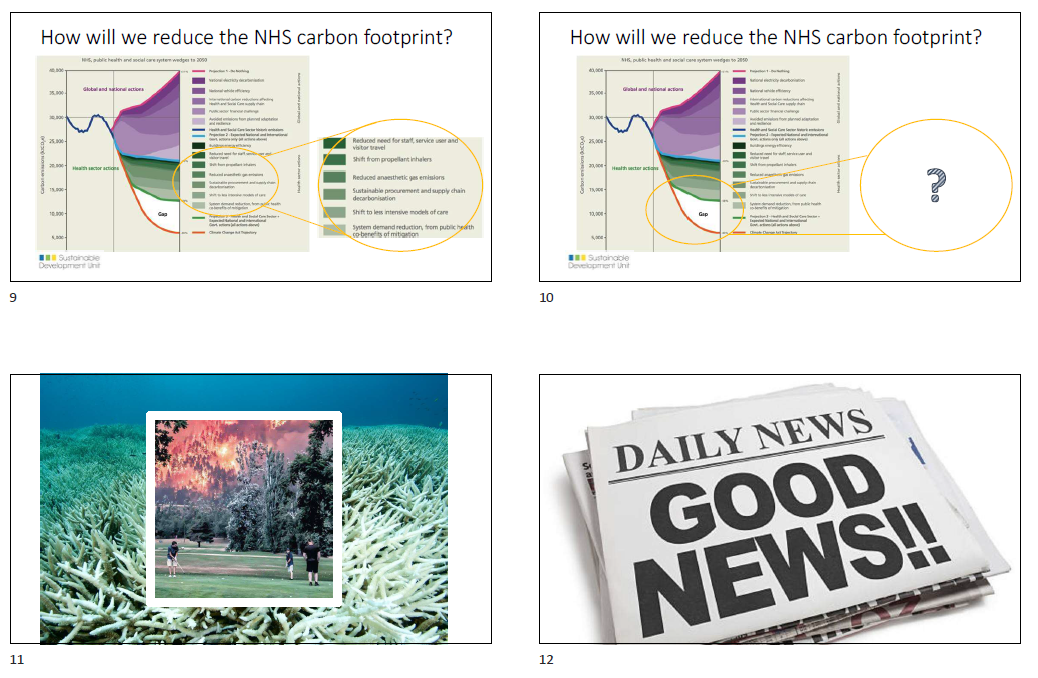


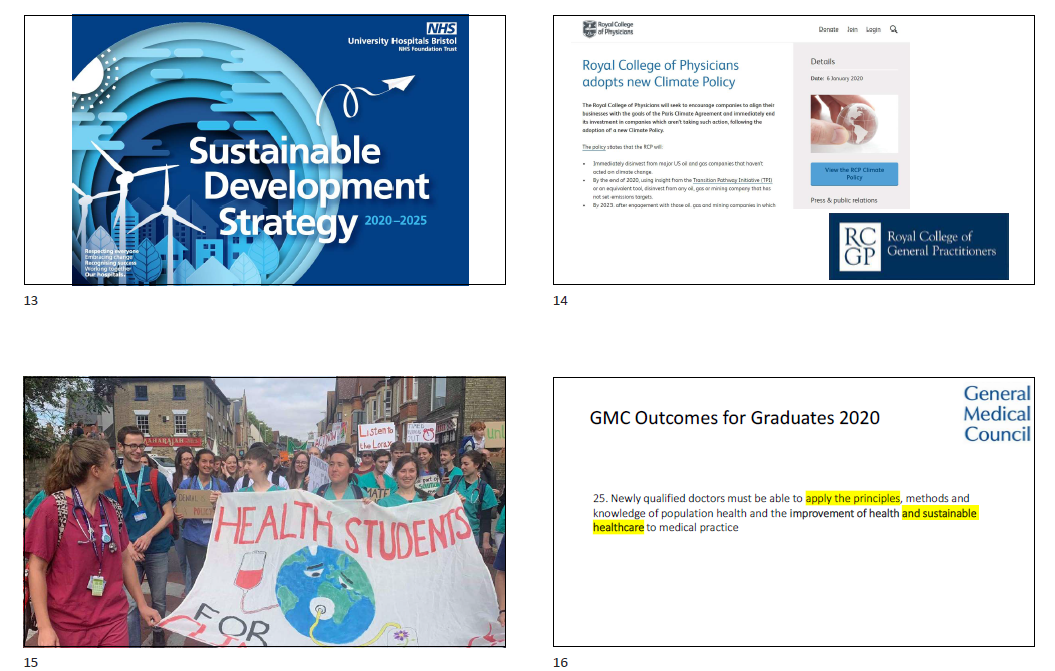


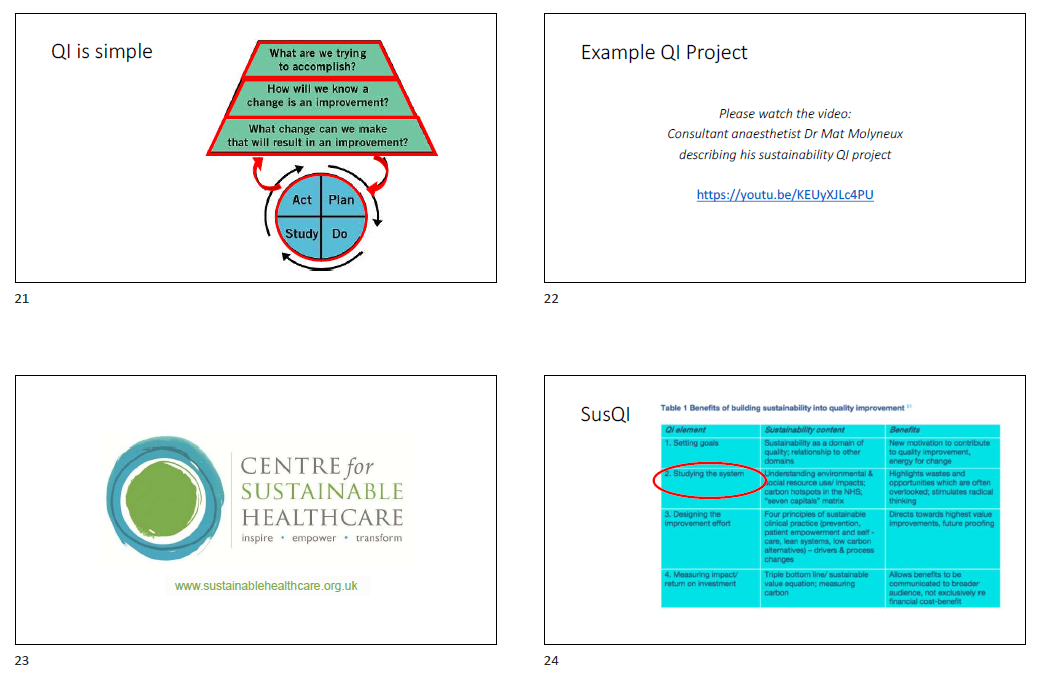

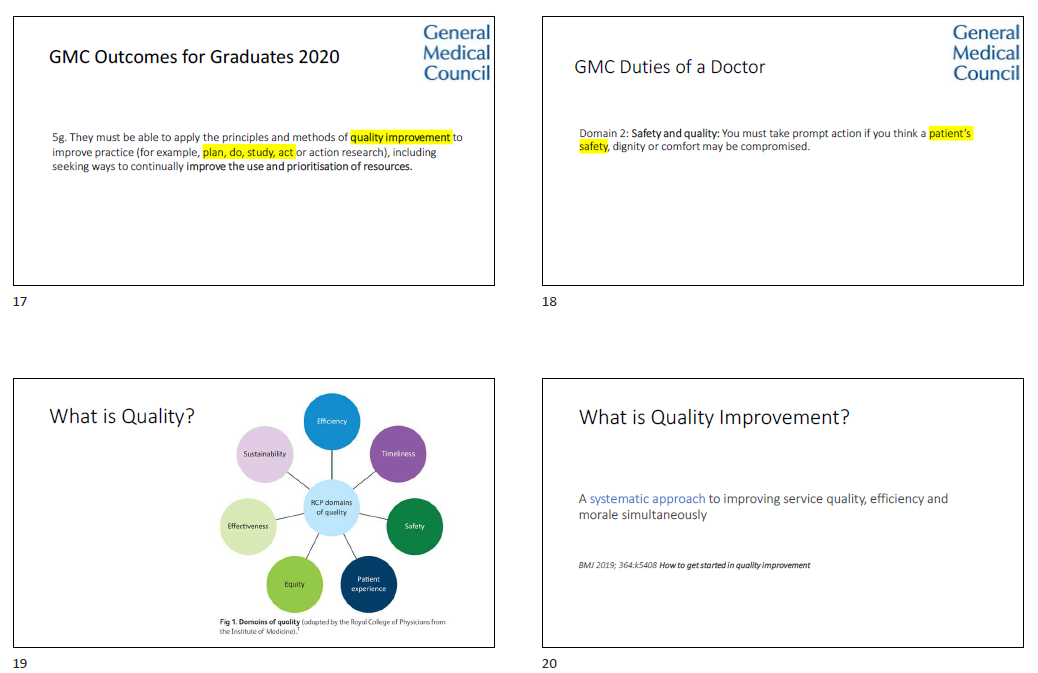


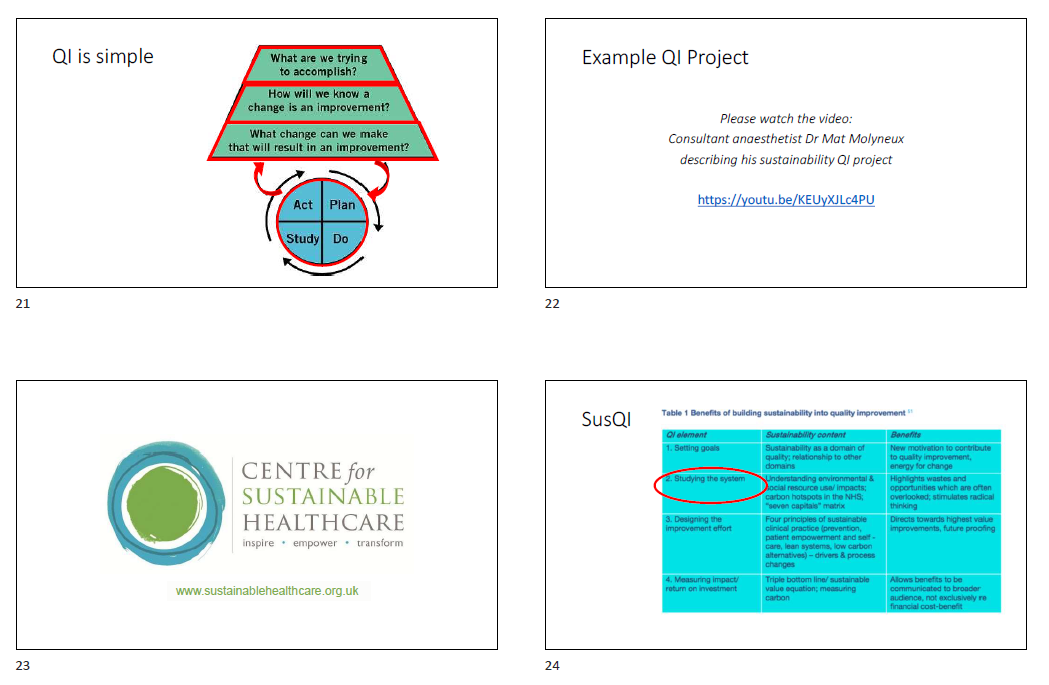


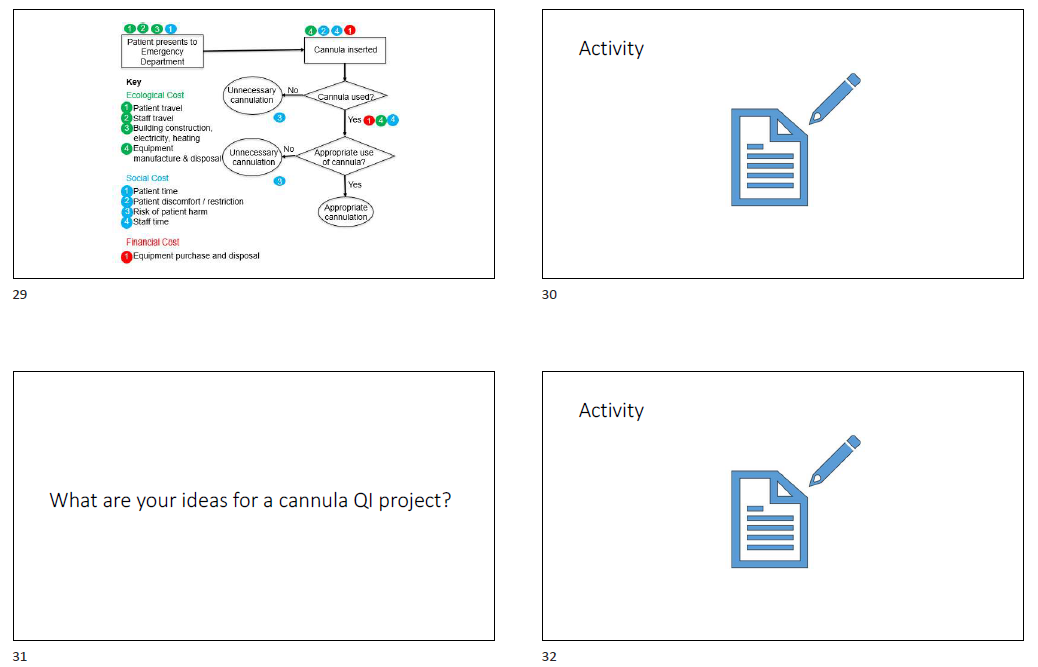

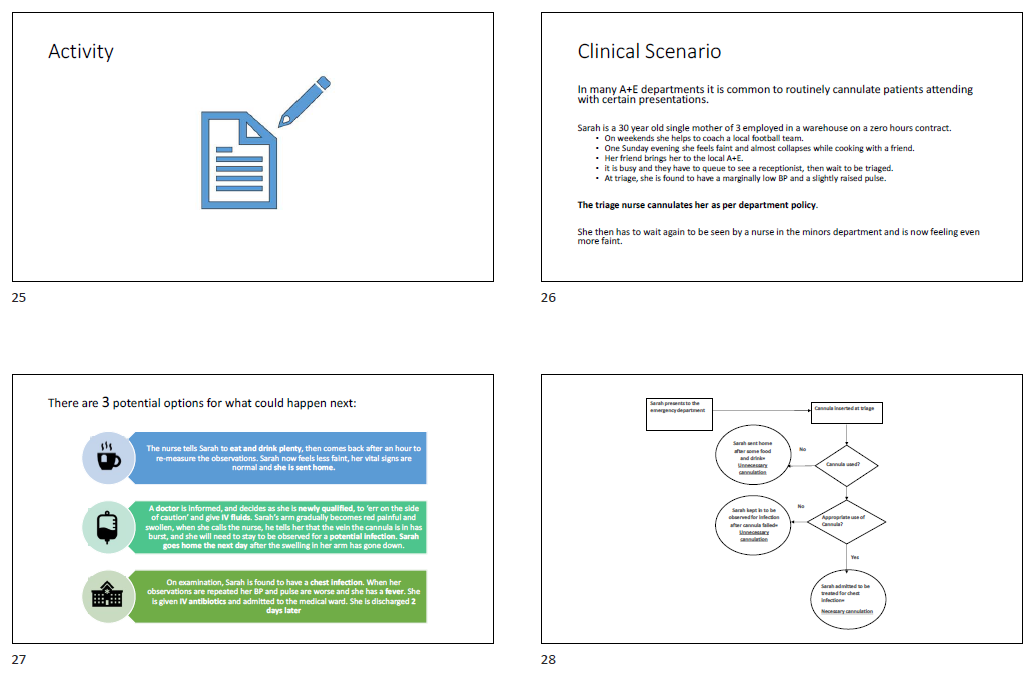


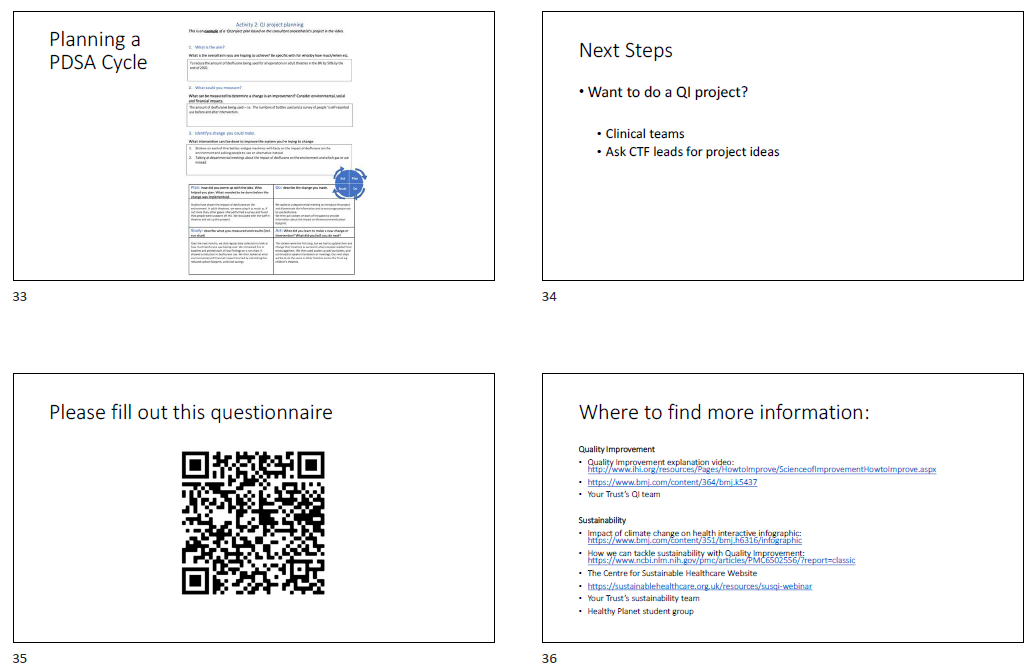


*Workshop Activity 1:* Consider the environmental, financial and social impacts of this cannulation pathway and then write them down next to the relevant stage of the pathway.

**
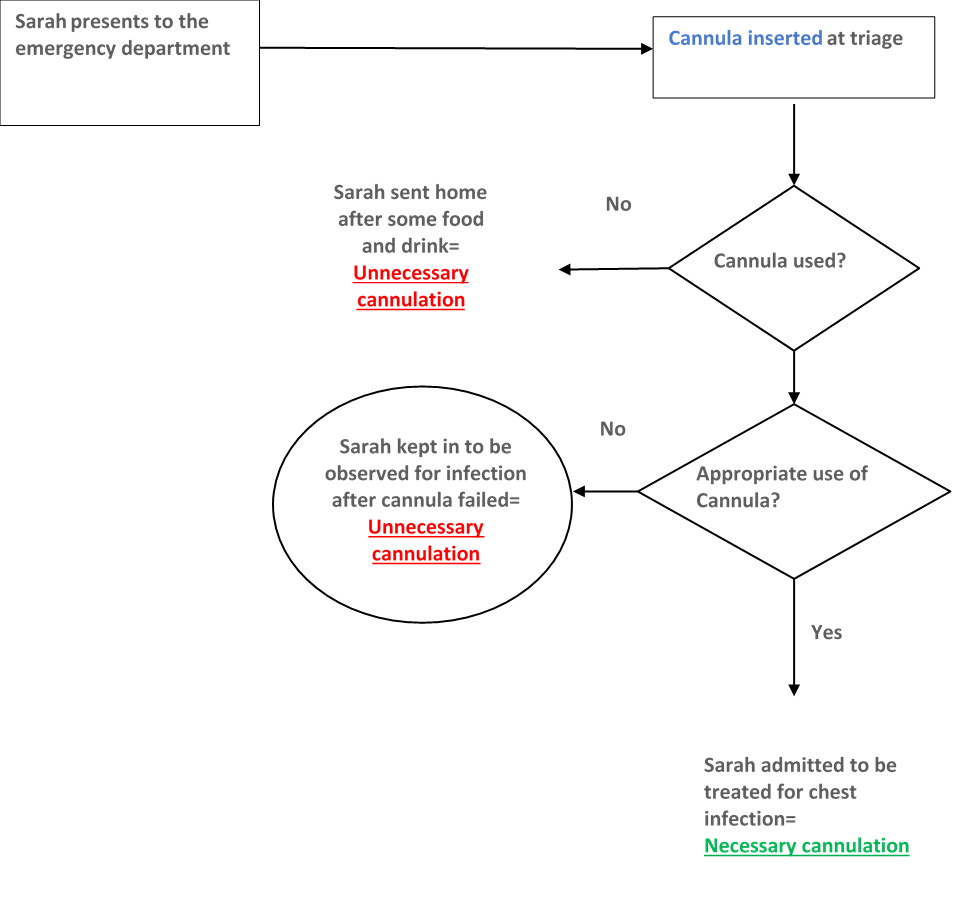
**

*Workshop Activity 2:* Using the SusQI project idea, use the following PDSA project plan template to outline your project plan

**
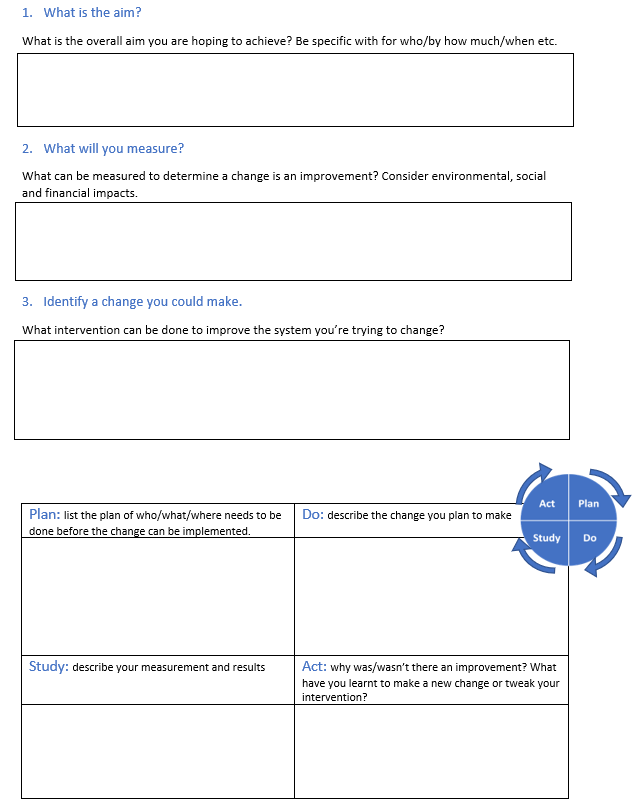
**
