# Supplementary material for: Sustainability in quality improvement (SusQI): a case-study in undergraduate medical education
Source: BMC Med Educ. 2021 Aug 12;21:425. doi: 10.1186/s12909-021-02817-2 (PMC8358256; doi:10.1186/s12909-021-02817-2)
Supplement: Supplementary file 2 — Additional file 2. [file 12909_2021_2817_MOESM2_ESM.docx]

**Sustainability in quality improvement (SusQI): a case-study in undergraduate medical education**

Philippa Clery^1,2^, Stuart d’Arch Smith^3^, Oliver Marsden^1^, Kathleen Leedham-Green^4^

^1^ Bristol Medical School, University of Bristol, Bristol, UK

^2^ University Hospitals Bristol and Weston NHS Foundation Trust, Bristol, UK

^3^ Centre for Sustainable Healthcare, Oxford, UK

^4^ Medical Education Research Unit, Imperial College London, London, UK

Pre and post-session questionnaire (questions in red were in post-questionnaire only)

**___________________________________________________________________________**

**Please rate your confidence in the following statements:**

**1** = not at all confident; **2** = a little confident; **3** = somewhat confident; **4** = fairly confident; **5** = very confident

1. I know what a quality improvement (QI) project involves^K,C^
2. I know how to identify when there is need for QI projects^K,C^
3. I can develop and undertake a QI project on my hospital placements^K,C^

**Please rate your knowledge of the following:**

**1** = very poor; **2** = poor; **3** = fair; **4** = good; **5** = excellent

1. The health impacts of climate change^K^
2. Sustainable healthcare^K^
3. Sustainability in quality improvement^K^

**Please rate the following statements:**

**1** = strongly disagree; **2** = disagree; **3** = unsure; **4** = agree; **5** = strongly agree

1. Quality improvement projects are important for improving patient care^A^
2. I am likely to be involved in QI projects in the future^A, Av^
3. It is important for quality improvement to be part of core teaching for medical students^A^
4. Quality improvement projects with a sustainability focus are important in the future of healthcare^A^
5. It is important for sustainable healthcare to be part of core teaching for medical students^A^
6. It is important for me to take actions to reduce carbon emissions in my future job^A^
7. I am likely to take action to reduce my environmental impact in my future job^A, Av^

**Please rate the following statement:**

How likely are you to take part in a quality improvement project following this session?

Very likely Likely Neutral Unlikely Very unlikely

**What suggestions do you have for future teaching on this topic?**
(Free text answers)

**Key**

^K^ = Knowledge, ^C^ = Confidence in skill , ^A^ = Attitudes , ^Av^ = Applied value
